# Supplementary material for: Napyradiomycin A4 and Its Relate Compounds, a New Anti-PRV Agent and Their Antibacterial Activities, from Streptomyces kebangsaanensis WS-68302
Source: Molecules. 2023 Jan 8;28(2):640. doi: 10.3390/molecules28020640 (PMC9861092; doi:10.3390/molecules28020640)
Supplement: Supplementary file 1 [file molecules-28-00640-s001.zip › molecules-2063603-supplementary.pdf]

Contents of Supporting Information

Contents

Figure S1 HR-ESI-MS spectrum of compound **1**	2
Figure S2 UPLC-MS total ion chromatogram of compound **1**	3
Figure S3 Electrospray mass spectrum of compound **1**	3
Figure S4 UV spectrum of compound **1**	4
Figure S5 IR spectrum of compound **1**	4
Figure S6 ^{1}H-NMR spectrum of compound **1** ($CDCl_3$, 700 MHz)	5
Figure S7 ^{13}C-NMR spectrum of compound **1** ($CDCl_3$, 175 MHz)	5
Figure S8 DEPT spectrum of compound **1**	6
Figure S9 HSQC spectrum of compound **1**	6
Figure S10 HMBC spectrum of compound **1**	7
Figure S11 ^{1}H–^{1}H COSY spectrum of compound **1**	7
Figure S12 NOESY spectrum of compound **1**	8
Figure S13 CD spectrum of compound **1**	8
Figure S14 CD spectrum of compound **3**	9
Figure S15 HR-ESI-MS spectrum of compound **2**	9
Figure S16 UPLC-MS total ion chromatogram of compound **2**	10
Figure S17 Electrospray mass spectrum of compound **2**	10
Figure S18 UV spectrum of compound **2**	11
Figure S19 ^{1}H-NMR spectrum of compound **2** ($CDCl_3$, 600 MHz)	11
Figure S20 ^{13}C-NMR spectrum of compound **2** ($CDCl_3$, 175 MHz)	12
Figure S21 DEPT spectrum of compound **2**	12
Figure S22 HSQC spectrum of compound **2**	13
Figure S23 HMBC spectrum of compound **2**	13
Figure S24 ^{1}H–^{1}H COSY spectrum of compound **2**	14
Figure S25 CD spectrum of compound **2**	14
Figure S26 CD spectrum of compound **5**	15

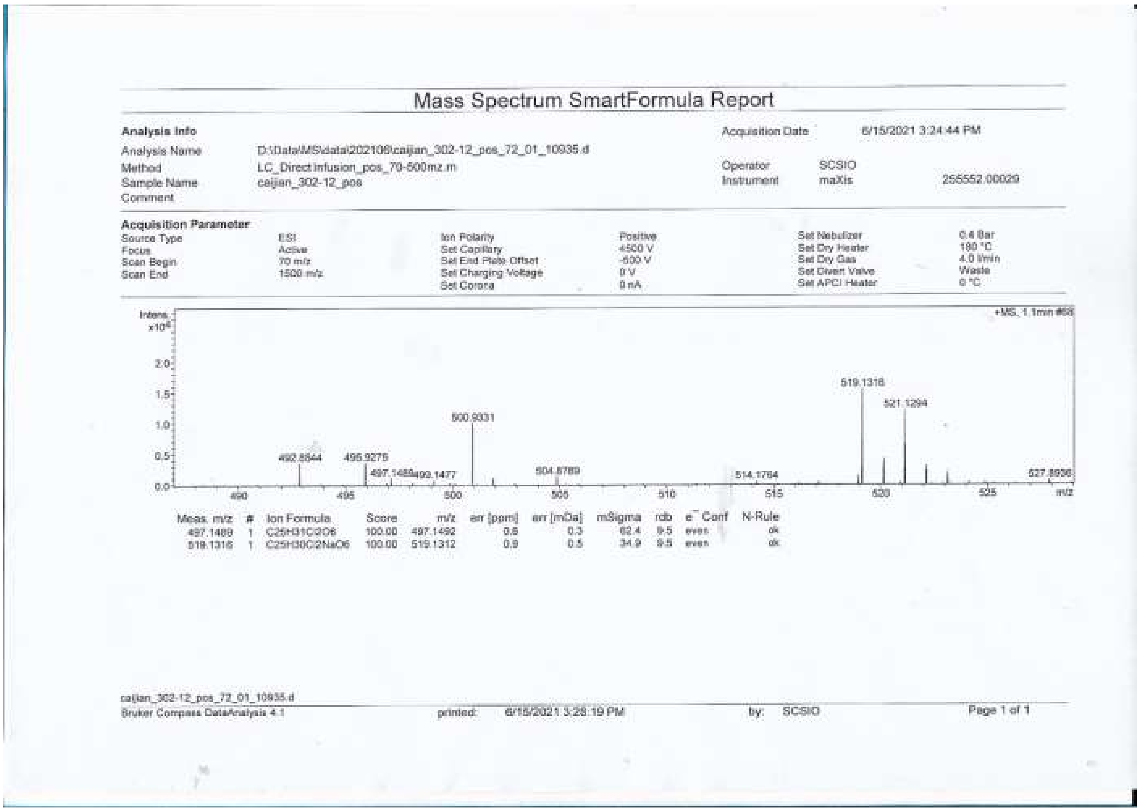

Figure S1. HR-ESI-MS spectrum of compound **1**

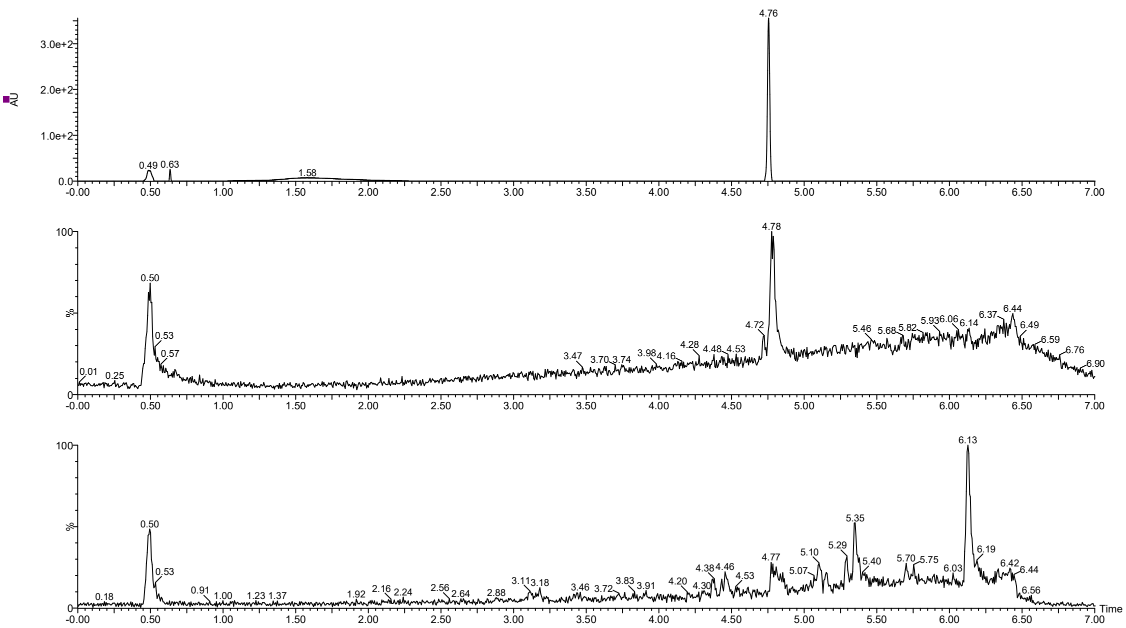

Figure S2. UPLC-MS total ion chromatogram of compound **1** (retention time 4.76min)

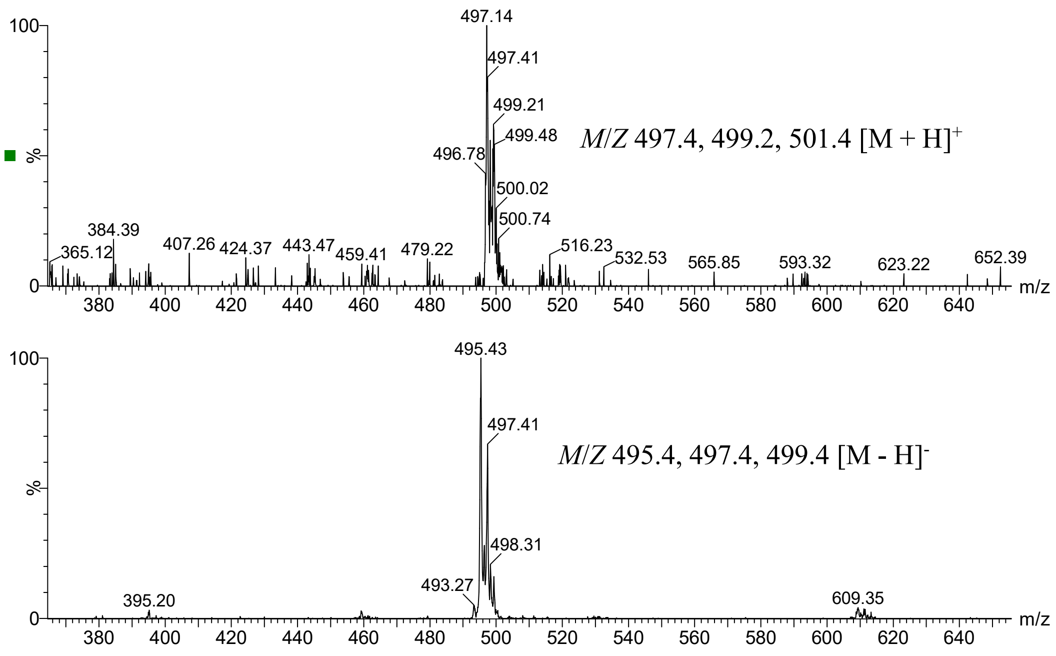

Figure S3. Electrospray mass spectrum of compound **1** (retention time 4.76 min)

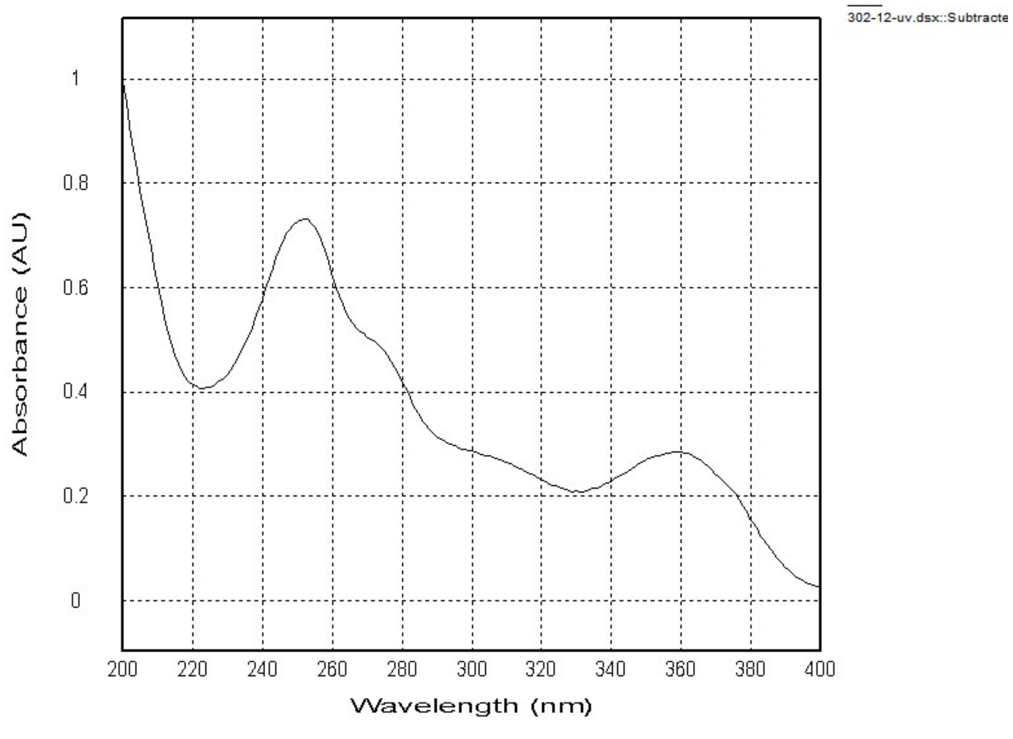

Figure S4. UV spectrum of compound **1**

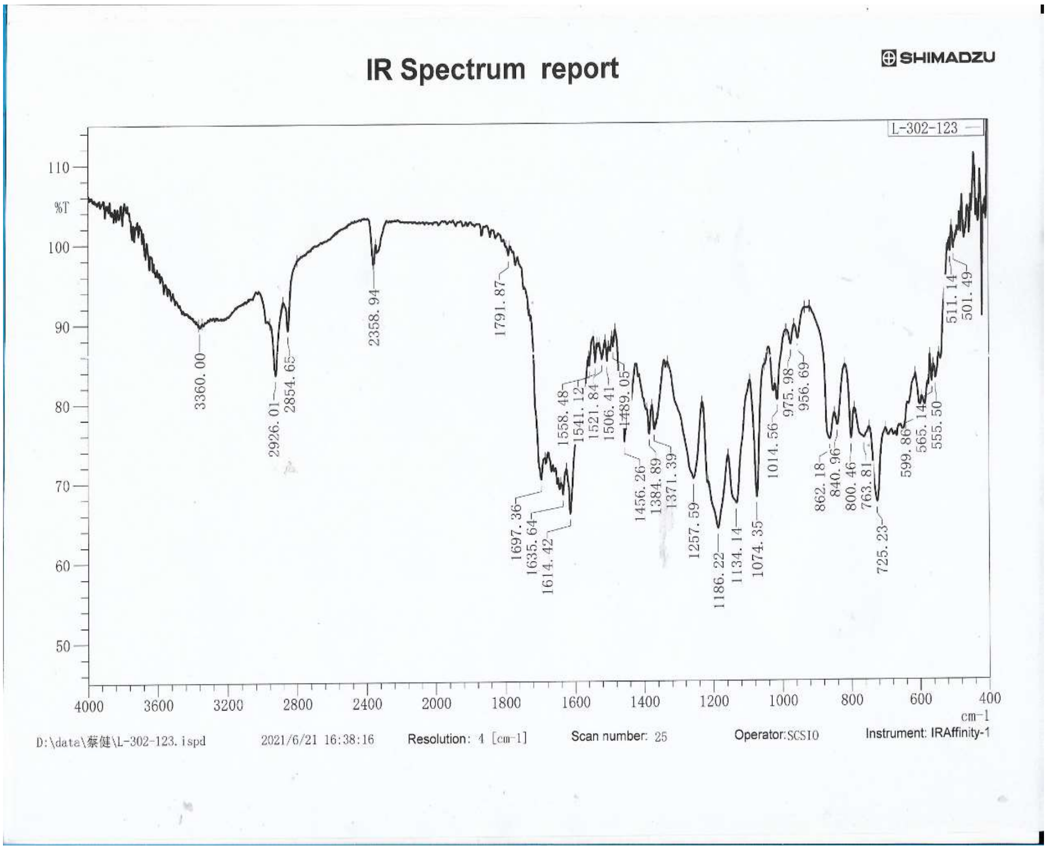

Figure S5. IR spectrum of compound **1**

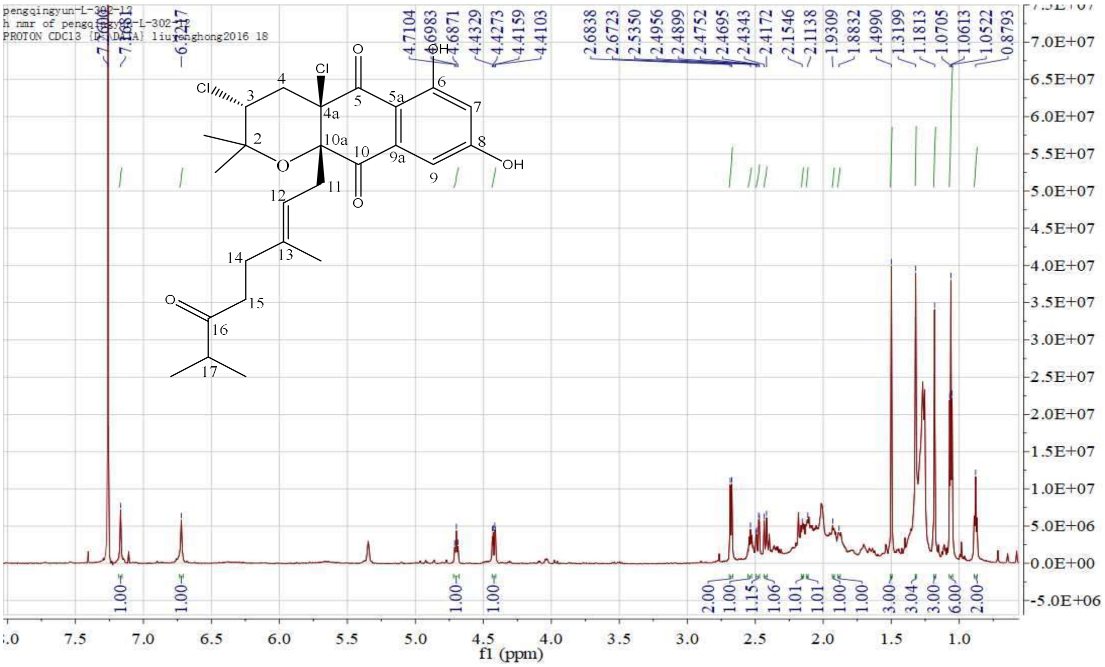

Figure S6. ^{1}H-NMR spectrum of compound **1** in $CDCl_3$

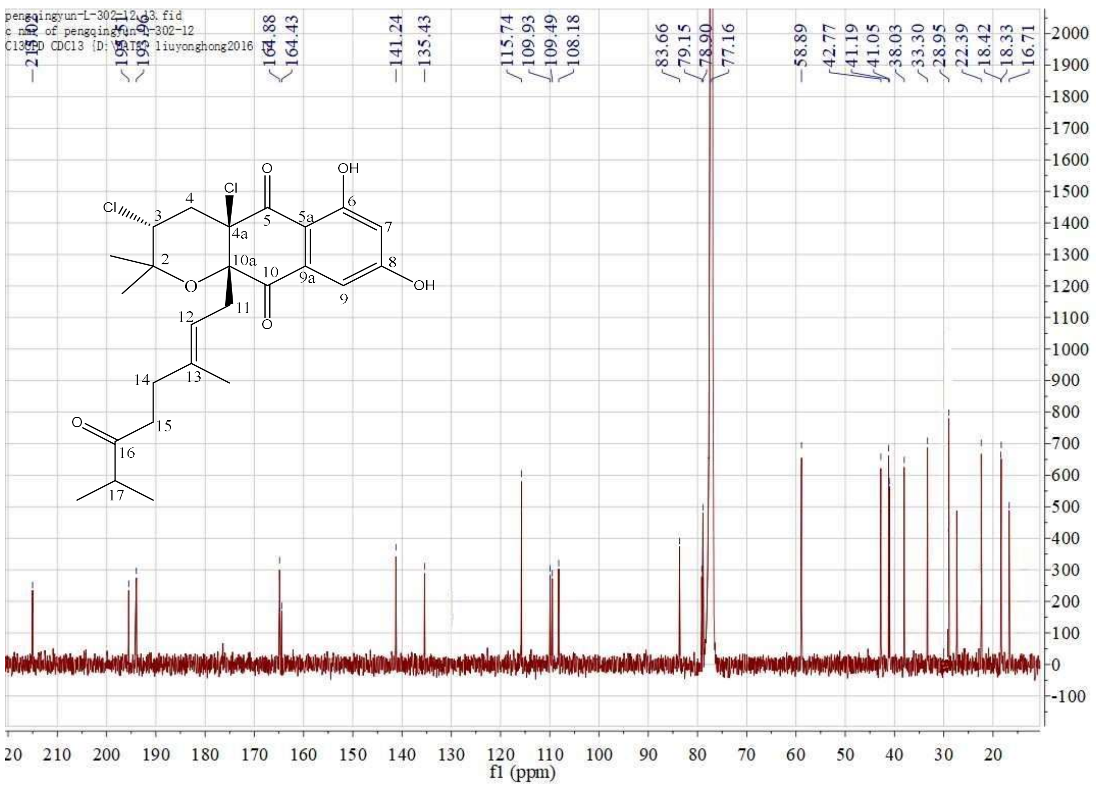

Figure S7. ^{13}C-NMR spectrum of compound **1** in $CDCl_3$

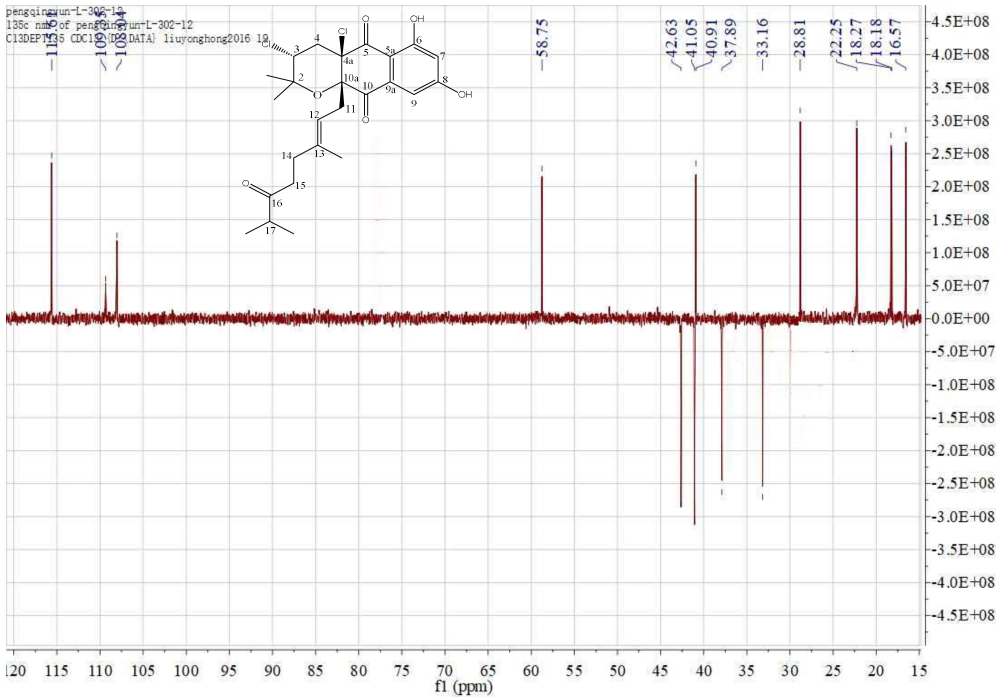

Figure S8. DEPT spectrum of compound **1** in $CDCl_3$

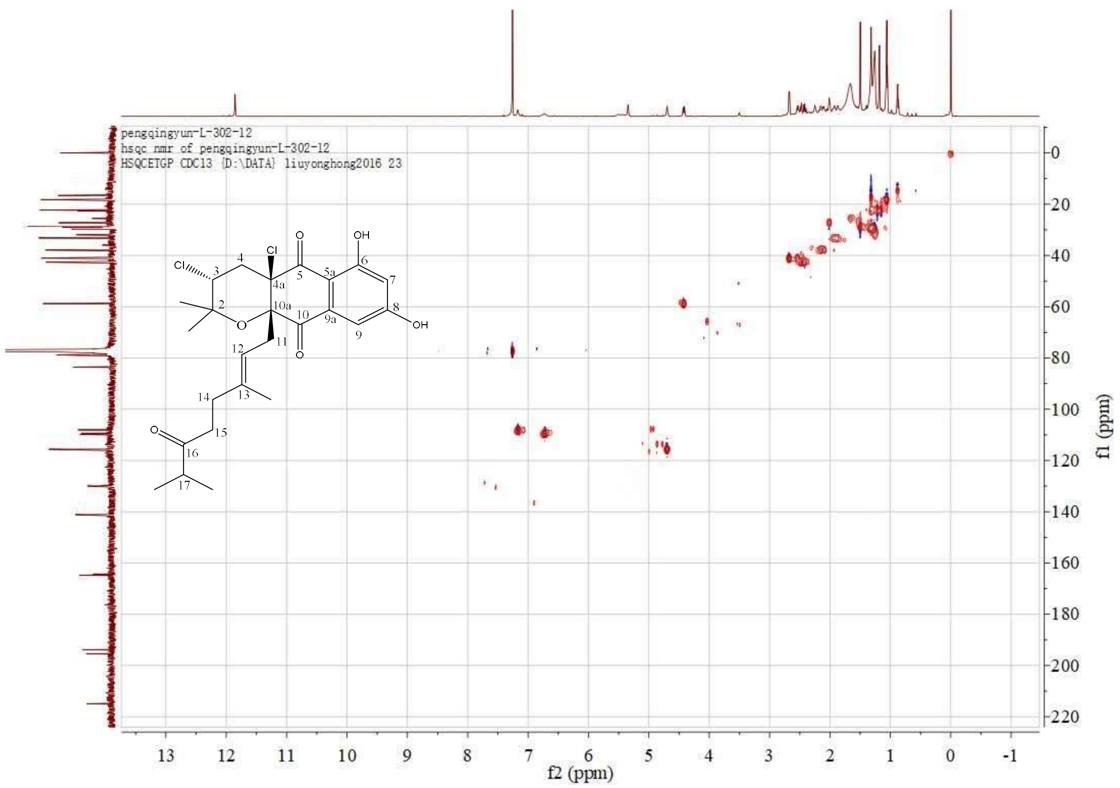

Figure S9. HSQC spectrum of compound **1** in $CDCl_3$

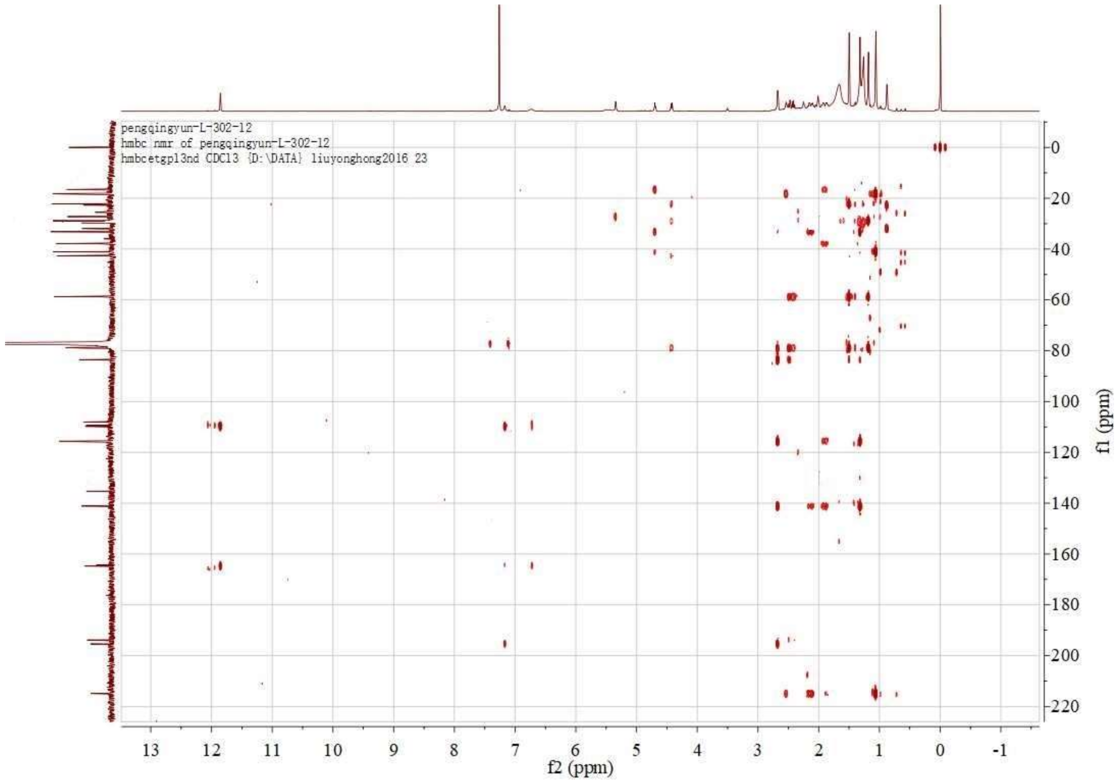

Figure S10. HMBC spectrum of compound **1** in $CDCl_3$

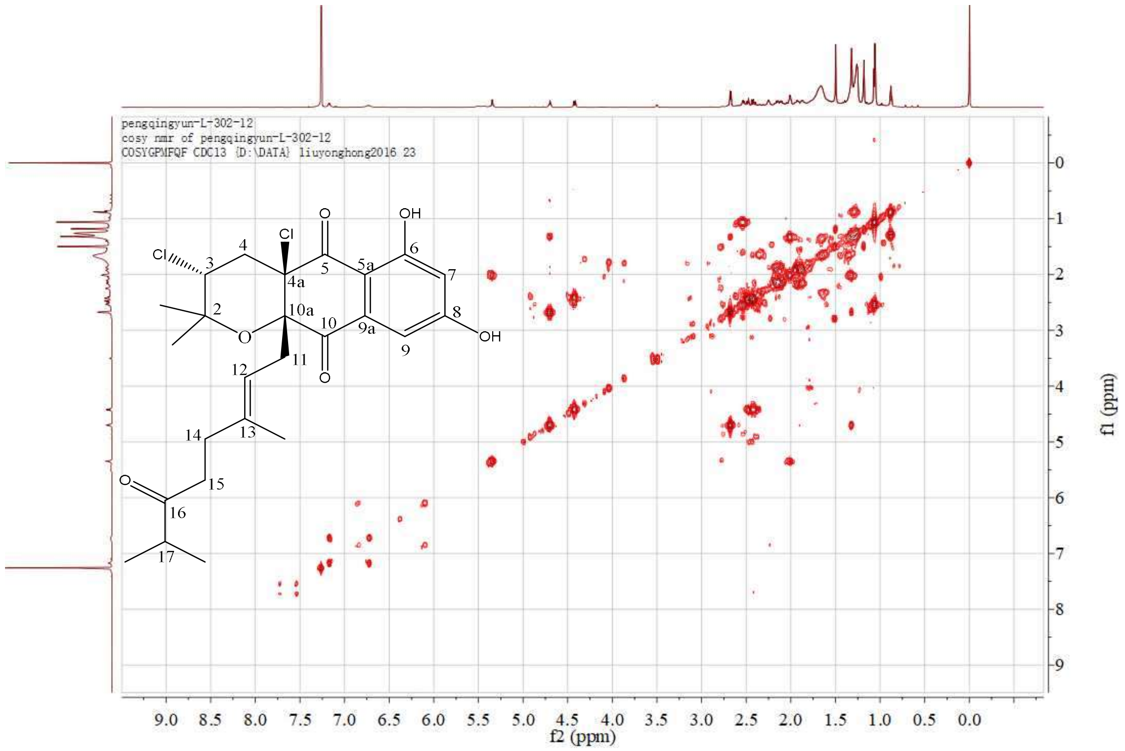

Figure S11. ^{1}H–^{1}H COSY spectrum of compound **1** in $CDCl_3$

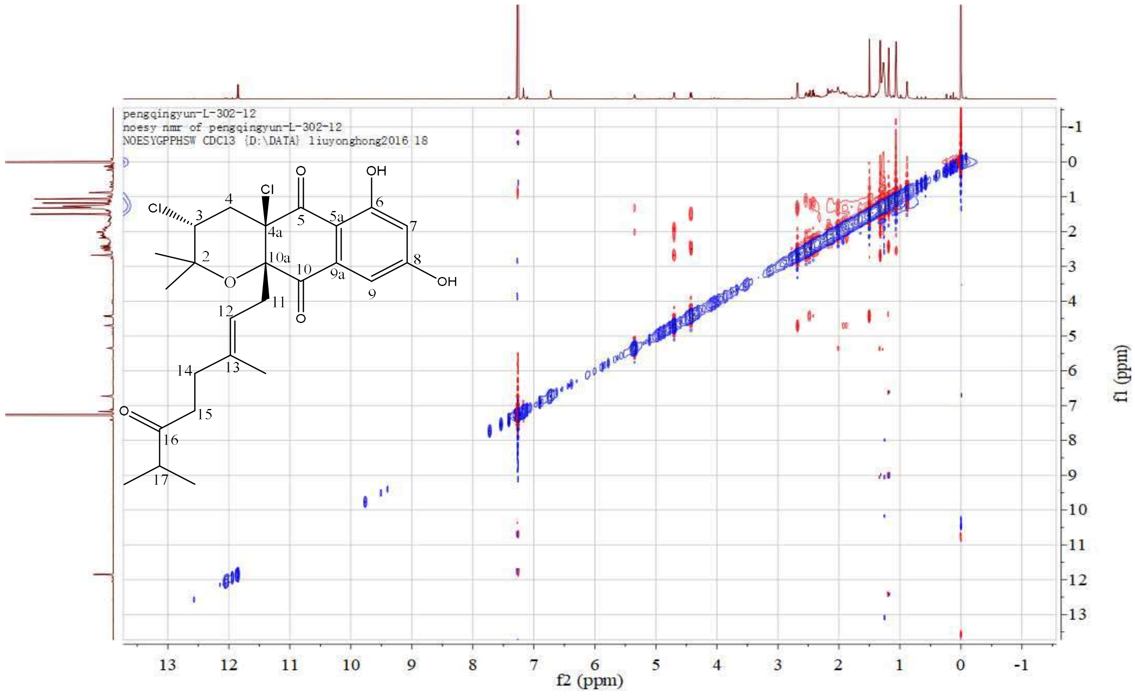

Figure S12. NOESY spectrum of compound **1** in $CDCl_3$

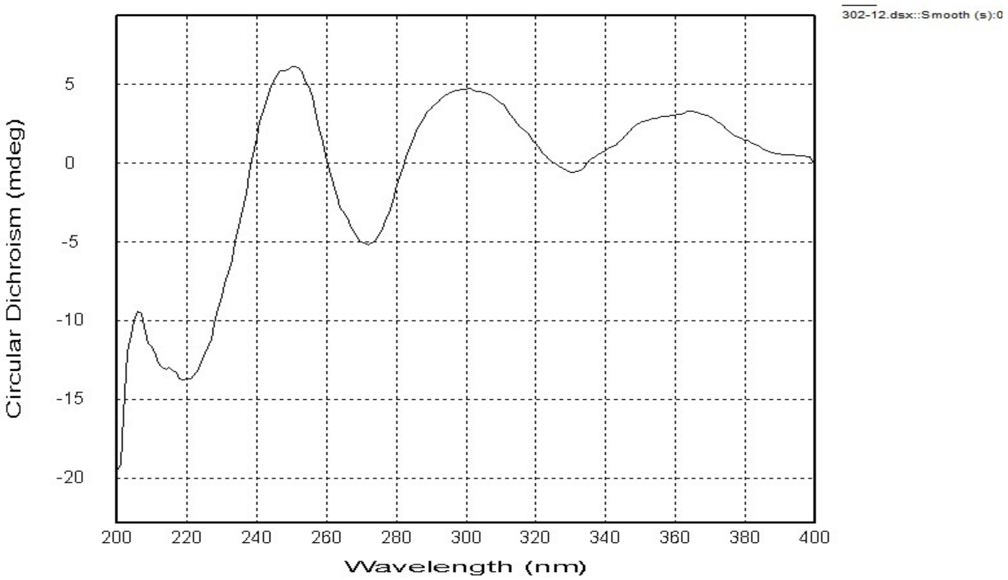

Figure S13. CD spectrum of compound **1**

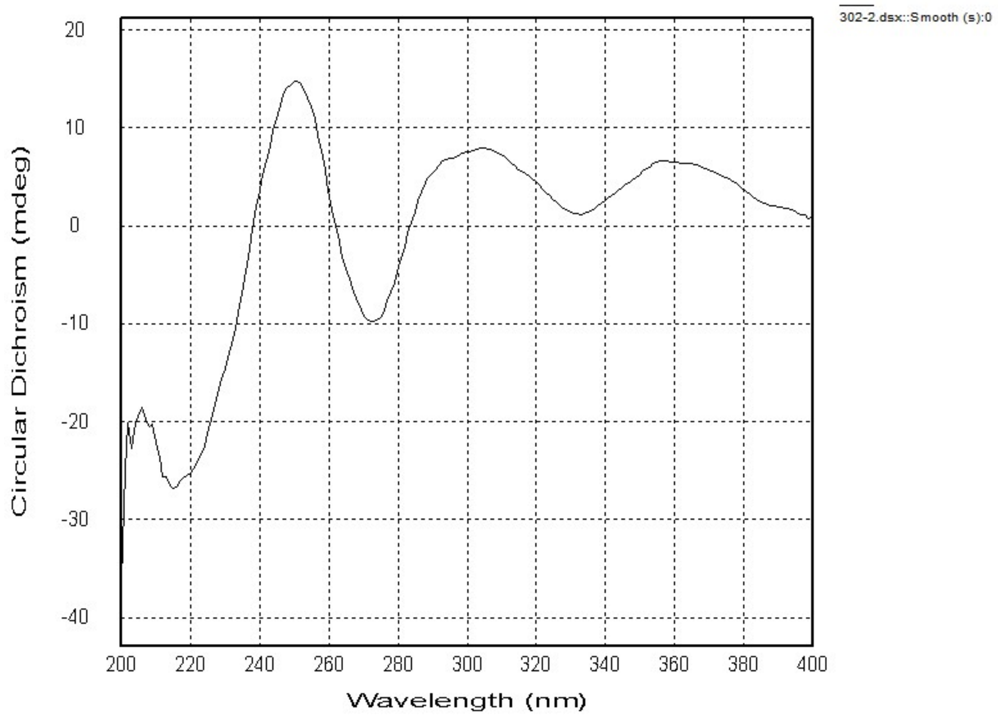

Figure S14. CD spectrum of compound **3**

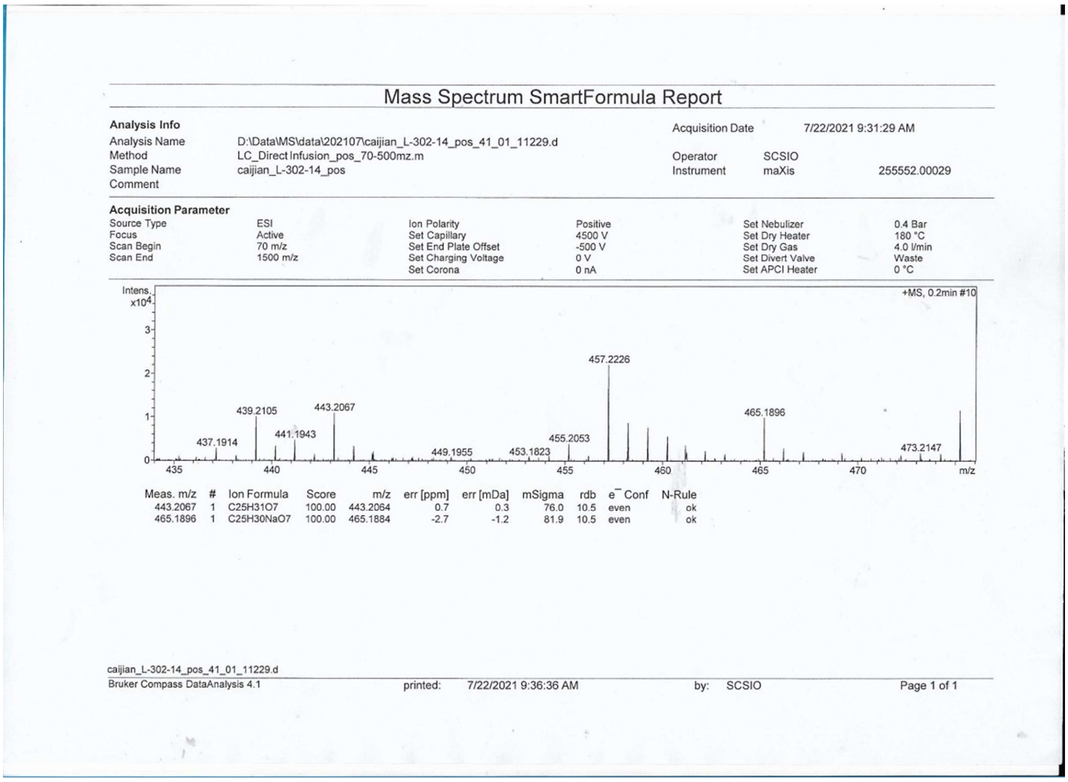

Mass Spectrum SmartFormula Report

Analysis Info		Acquisition Date	7/22/2021 9:31:29 AM
Analysis Name	D:\Data\MS\data\202107\caijian_L-302-14_pos_41_01_11229.d		
Method	LC_Direct Infusion_pos_70-500mz.m	Operator	SCSIO
Sample Name	caijian_L-302-14_pos	Instrument	maXis 255552.00029
Comment			

Acquisition Parameter

Source Type	ESI	Ion Polarity	Positive	Set Nebulizer	0.4 Bar
Focus	Active	Set Capillary	4500 V	Set Dry Heater	180 °C
Scan Begin	70 m/z	Set End Plate Offset	-500 V	Set Dry Gas	4.0 l/min
Scan End	1500 m/z	Set Charging Voltage	0 V	Set Divert Valve	Waste
		Set Corona	0 nA	Set APCI Heater	0 °C

Meas. m/z	#	Ion Formula	Score	m/z	err [ppm]	err [mDa]	mSigma	rdb	e⁻ Conf	N-Rule
443.2067	1	C25H31O7	100.00	443.2064	0.7	0.3	76.0	10.5	even	ok
465.1896	1	C25H30NaO7	100.00	465.1884	-2.7	-1.2	81.9	10.5	even	ok

caijian_L-302-14_pos_41_01_11229.d
Bruker Compass DataAnalysis 4.1 printed: 7/22/2021 9:36:36 AM by: SCSIO Page 1 of 1

Figure S15. HR-ESI-MS spectrum of compound **2**

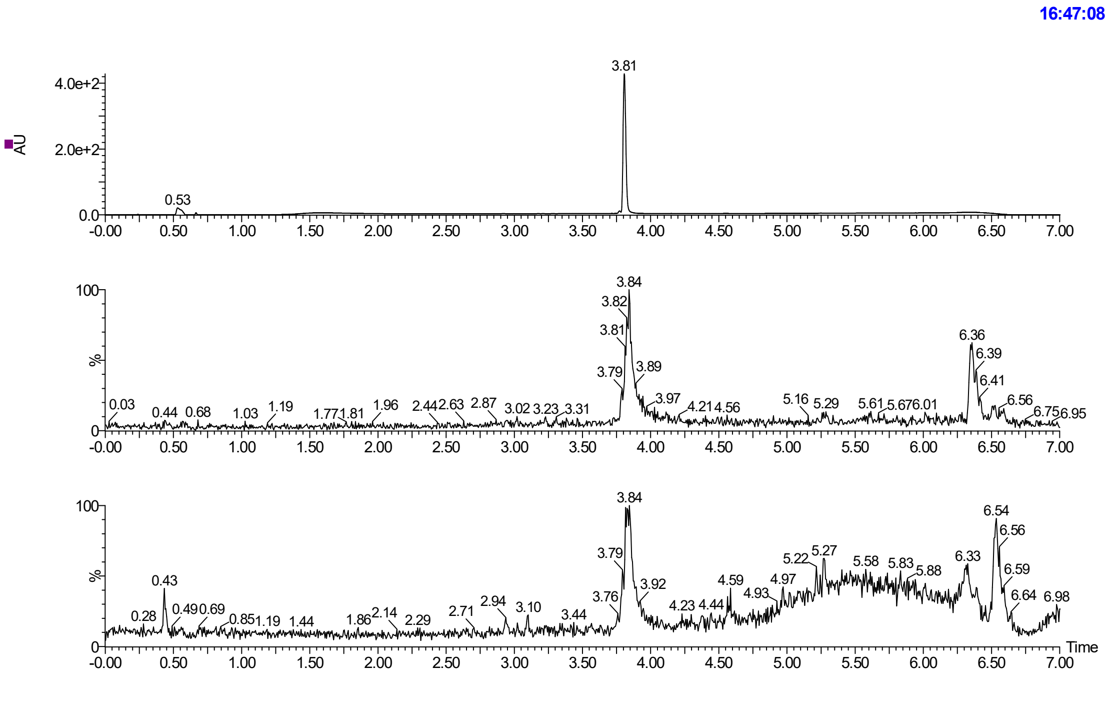

Figure S16. UPLC-MS total ion chromatogram of compound **2** (retention time 3.81 min)

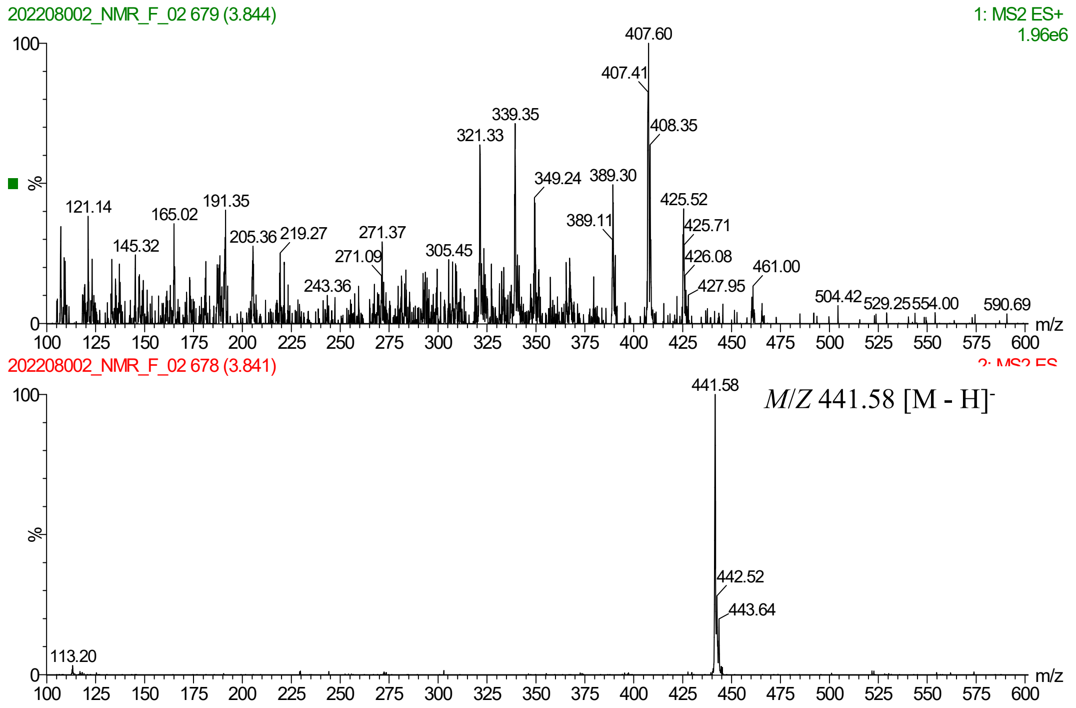

Figure S17. Electrospray mass spectrum of compound **2** (retention time 3.81 min)

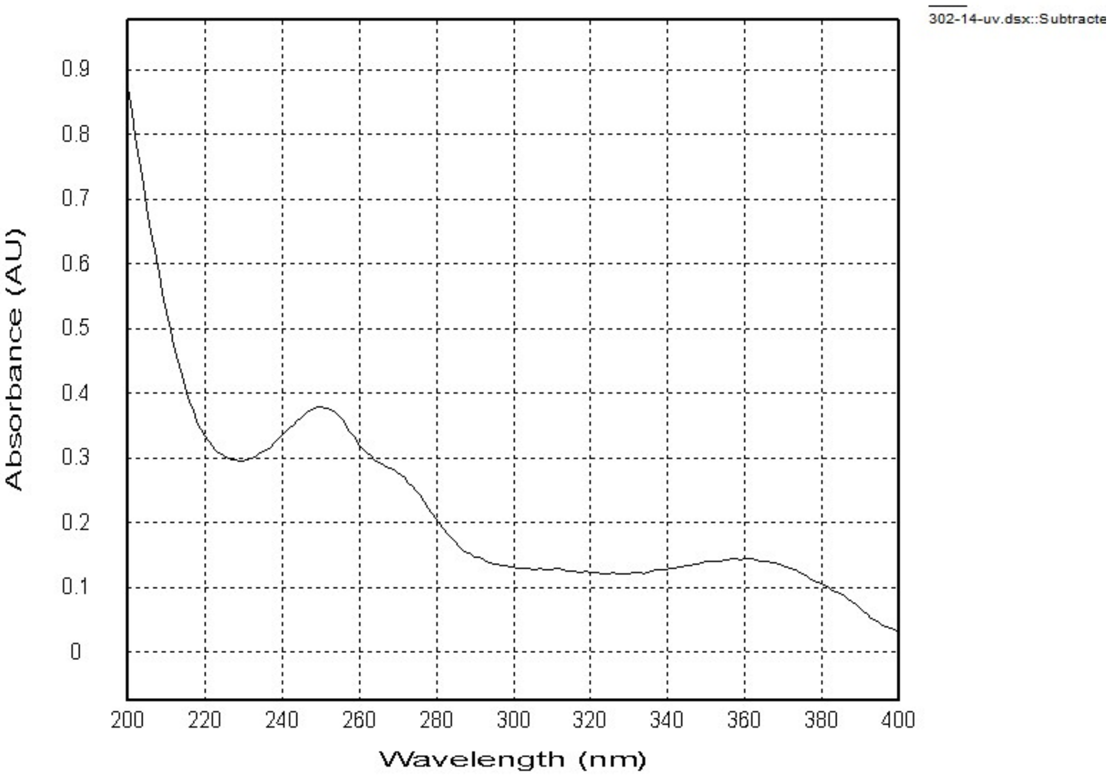

Figure S18. UV spectrum of compound **2**

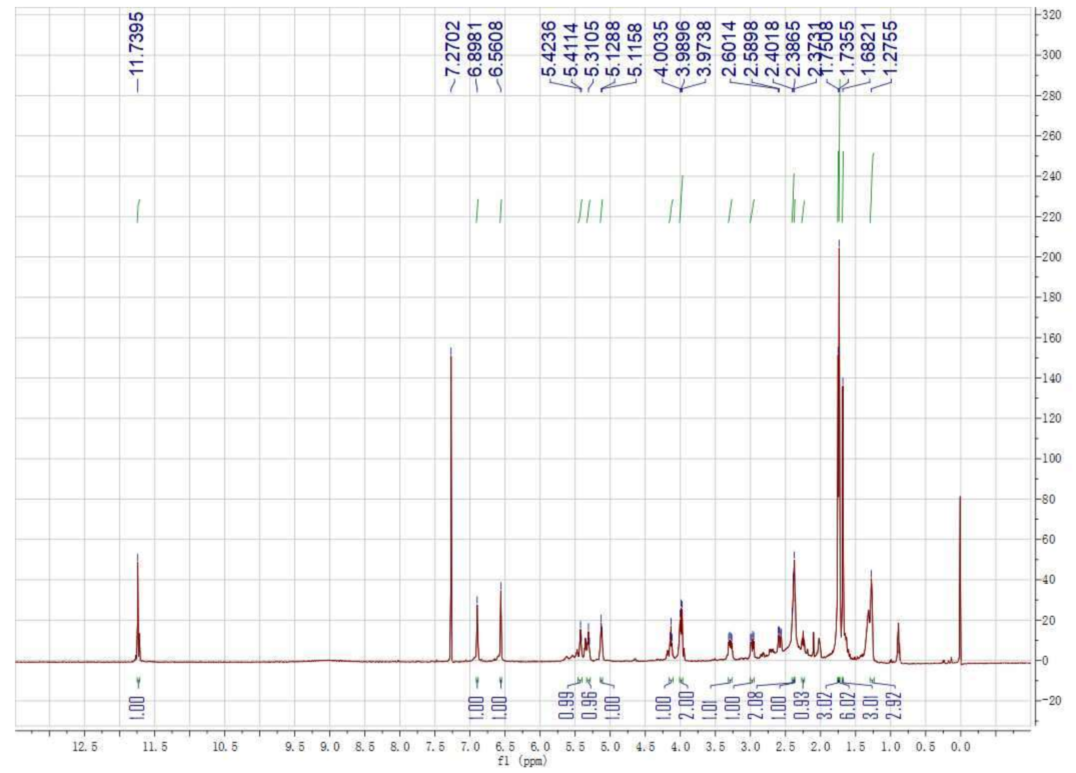

Figure S19. ^{1}H-NMR spectrum of compound **2** in $CDCl_3$

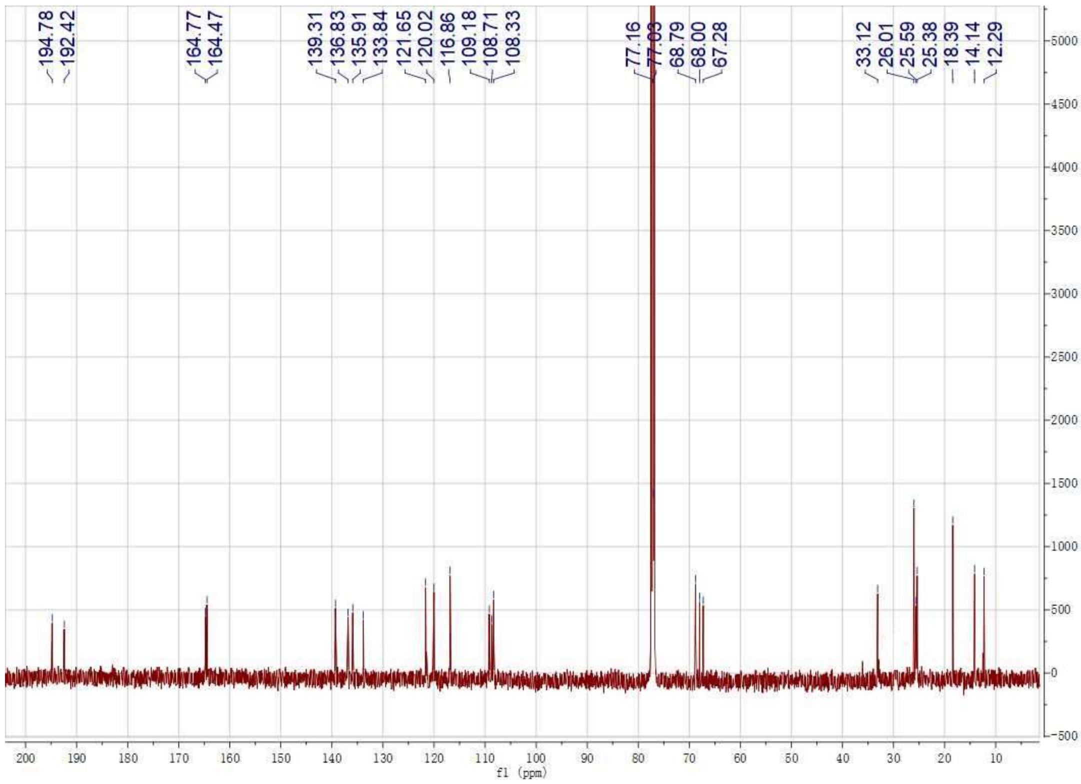

Figure S20. ^{13}C-NMR spectrum of compound **2** in $CDCl_3$

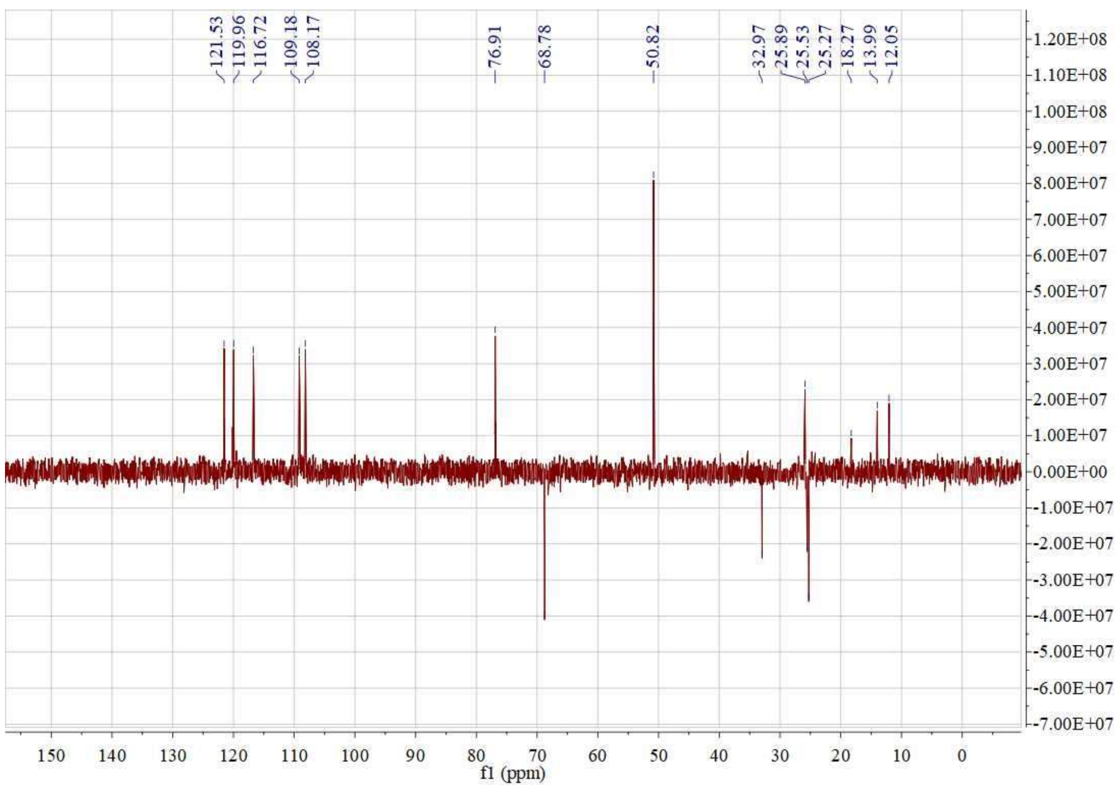

Figure S21. DEPT spectrum of compound **2** in $CDCl_3$

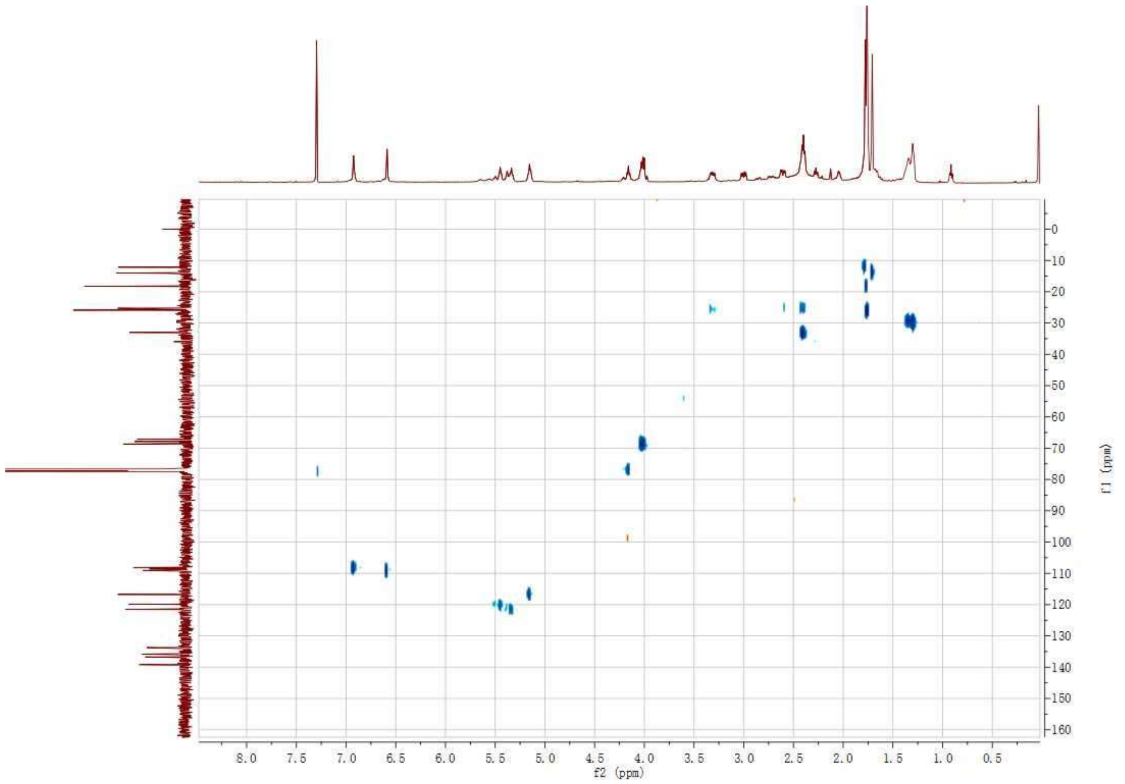

Figure S22. HSQC spectrum of compound **2** in $CDCl_3$

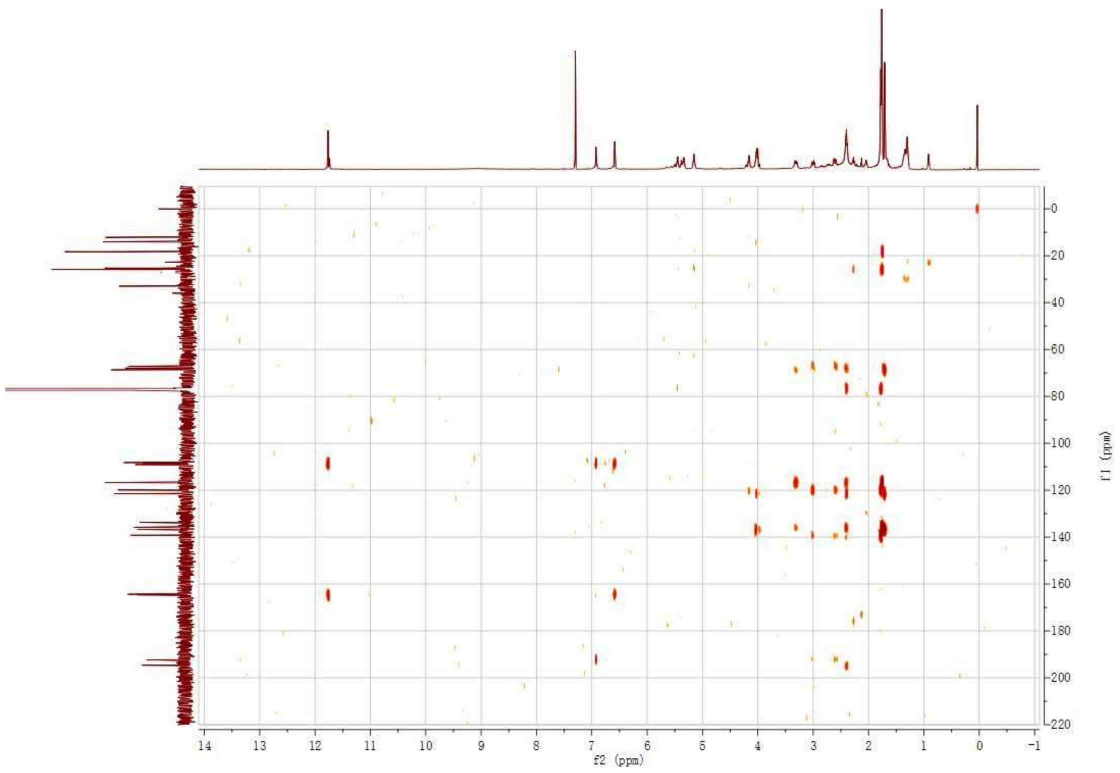

Figure S23. HMBC spectrum of compound **2** in $CDCl_3$

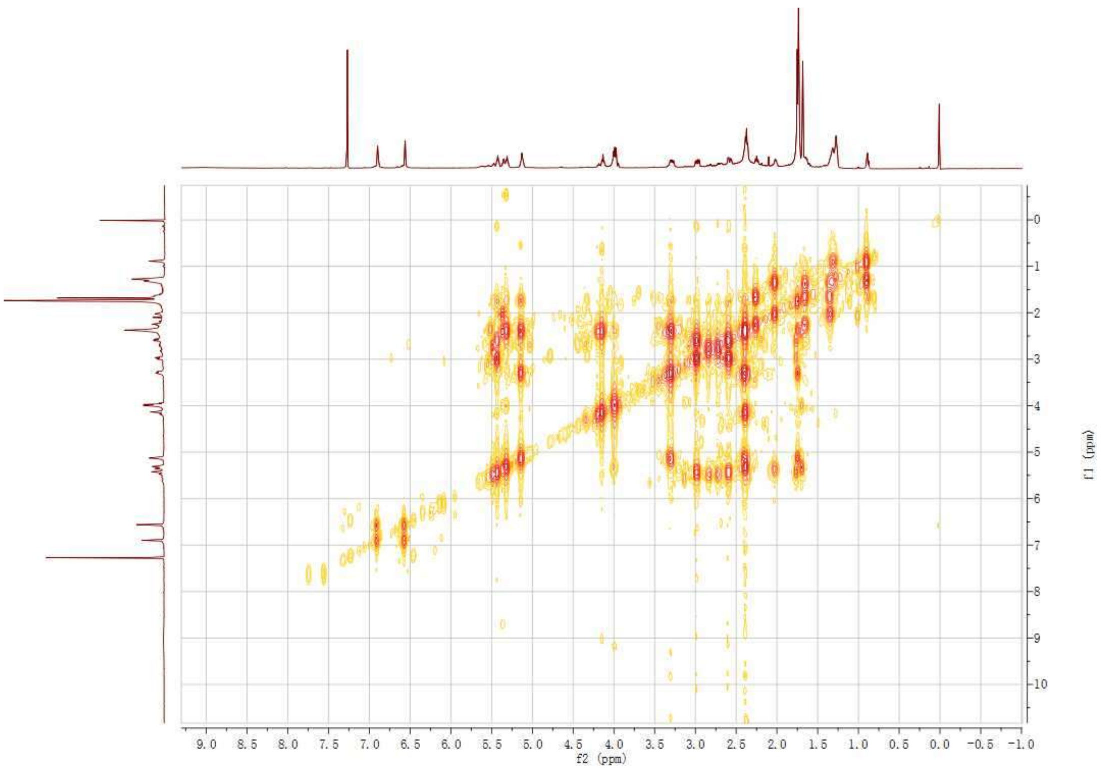

Figure S24. ^1H-^1H COSY spectrum of compound **2** in $CDCl_3$

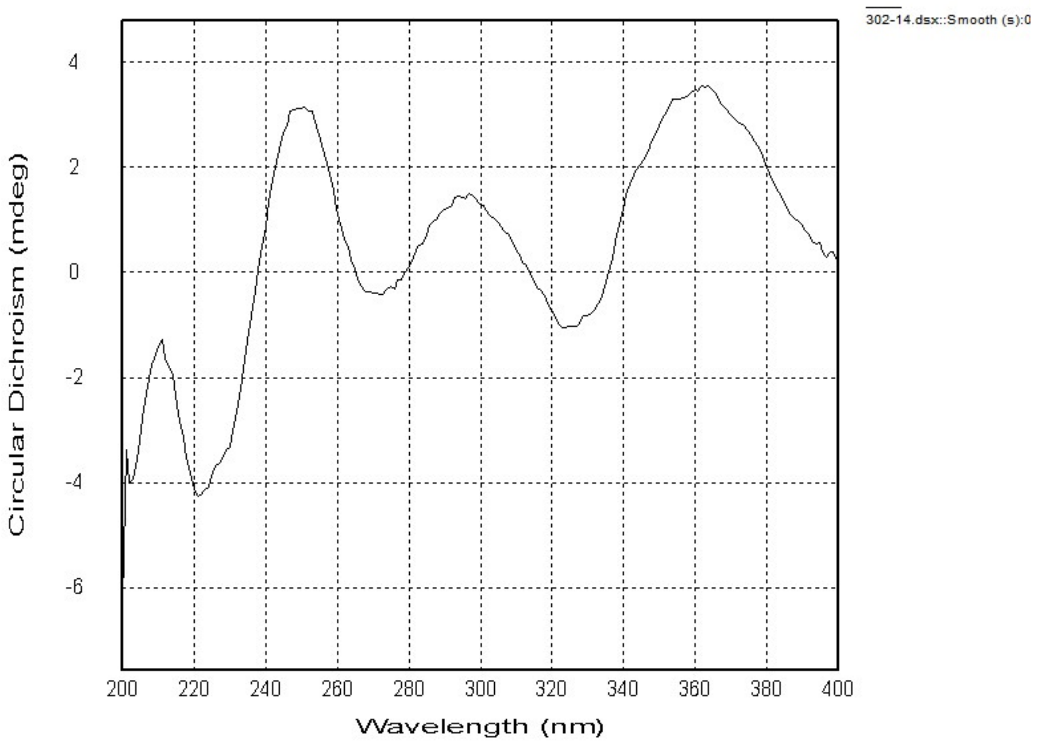

Figure S25. CD spectrum of compound **2**

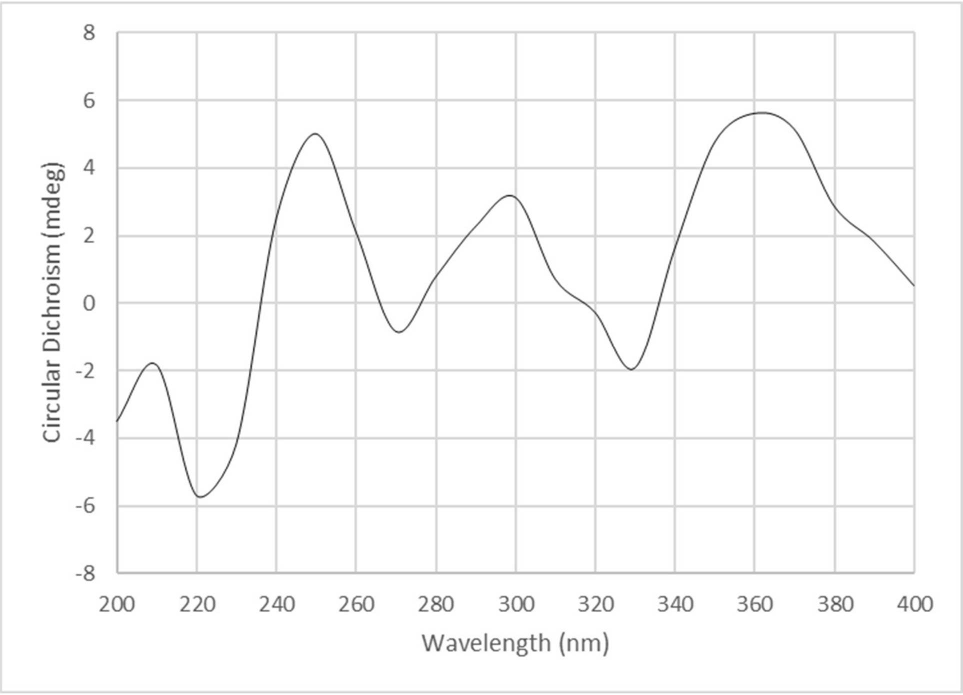

Figure S26. CD spectrum of compound **5**
